# Association between Work-Family Conflict and Depressive Symptoms among Chinese Female Nurses: The Mediating and Moderating Role of Psychological Capital

**DOI:** 10.3390/ijerph120606682

**Published:** 2015-06-12

**Authors:** Junhui Hao, Di Wu, Li Liu, Xirui Li, Hui Wu

**Affiliations:** 1 Department of Social Medicine, School of Public Health, China Medical University, No.77 Puhe Road, Shenyang North New Area, Shenyang 110013, China; E-Mails: blink007@sina.cn (J.H.); liul@mail.cmu.edu.cn (L.L.); 2 Department of English, School of Basic Medicine, China Medical University, No.77 Puhe Road, Shenyang North New Area, Shenyang 110013, China; E-Mails: freebirdie96@126.com (D.W.); lixirui1004@sina.cn (X.L.)

**Keywords:** depressive symptoms, work-family conflict, psychological capital

## Abstract

Depressive symptoms have been in the limelight for many kinds of people, but few studies have explored positive resources for combating depressive symptoms among Chinese nurses. The purpose of this study is to explore the association between work-family conflict (WFC) and depressive symptoms among Chinese female nurses, along with the mediating and moderating role of psychological capital (PsyCap) in this relationship. This cross-sectional study was completed during the period of September and October 2013. A questionnaire that consisted of the Center for Epidemiologic Studies Depression Scale, the Work-Family Conflict scale and the Psychological Capital Questionnair scale was distributed to nurses in Shenyang, China. A total of 824 individuals (effective response rate: 74.9%) participated. Asymptotic and resampling strategies explored the mediating role of PsyCap in the relationship between WFC and depressive symptoms. Hierarchical linear regression analyses were performed to explore the moderating role of PsyCap. Both WFC and family-work conflict (FWC) were positively related with depressive symptoms. PsyCap positively moderated the relationship of WFC with depressive symptoms. Self-efficacy and hope positively moderated the relationship of WFC with depressive symptoms. PsyCap partially mediated the relationship of FWC with depressive symptoms. Hope and optimism partially mediated the relationship of FWC with depressive symptoms. Work-family conflict, as the risk factor of depressive symptoms, can increase nurses’ depressive symptoms, and PsyCap is a positive resource to combat nurses’ depressive symptoms. PsyCap can aggravate the effects of WFC on depressive symptoms and FWC can impact PsyCap to increase nurses’ depressive symptoms.

## 1. Introduction

Depressive symptoms have been in the limelight around the world. Depression has affected people from all walks of life, such as employees, college students, nurses and so on [1,2,3]. Depression not only affects people’s quality of life, but it also affects workplace productivity and can lead to direct economic costs [4,5]. Nurses are considered an at-risk population for depressive symptoms [6,7]. As an indispensable part of the workforce in the healthcare system, nurses work at hospitals under immense physical and psychological pressure. The negative psychosocial factors in the job environment are linked to underdeveloped psychological and physical health in nurses of some countries. Meanwhile, depressive symptoms among nurses affect the quality of their work, which have an influence on patients’ health. It is reported that depressive symptoms of nurses were related to the perception of lower patient safety [6]. In China, the proportion of nurses to the general population is 1:1750, which is lower than those of developed countries (1:140–1:320) and the majority of countries in the world (1:330) [8,9]. The huge population and the shortage of nurses is more likely to trigger depressive symptoms among Chinese nurses. Therefore, exploring the risk factors of nurses’ depressive symptoms is crucial to improve nurses’ health, and more importantly, increase the quality of health care services in China. 

Among occupational groups, some social and psychological outcomes have been identified as risk factors for depressive symptoms [6,10]. Work-family conflict (WFC) is a bidirectional conflict that includes both WFC and family-work conflict (FWC) [11,12,13]. WFC is “a form of inter role conflict in which the general demands, time devoted to and strain created by the job interfere with performing family-related responsibilities,” and FWC is “a form of inter role conflict in which the general demands, time devoted to and strain created by the family interfere with performing work-related responsibilities”. WFC and FWC have been found to be positively related to depressive symptoms in previous studies [10,14]. For nurses, the previous study shows that WFC had a significant connection to psychological health [15]. In Japan, the study shows that WFC had an impact on the physical and mental health of nurses [16].

In China, most families are dual-career couples. Compared with other occupational populations, nurses devote a great deal of time and energy to work and experience a higher level of WFC in China [17]. Although the association between WFC and depressive symptoms has been tested in other occupational populations [10,18,19], the association between WFC and depressive symptoms among Chinese female nurses is not clear. We will discuss whether WFC is the risk factor of depressive symptoms, and if controlling this risk factor can reduce nurses’ depressive symptoms. Thus, we have included WFC and FWC in our study and hypothesize that both WFC and FWC have associations with depressive symptoms among Chinese nurses.

It is widely acknowledged that positive organizational behavior can improve workers’ physical and mental health by strengthening the psychological resource of workers. Psychological capital (PsyCap) has been found as a positive resource for relieving depressive symptoms [20]. PsyCap is “a positive state of mind exhibited during the growth and development of an individual” and it includes the four core components of self-efficacy, optimism, resiliency, and hope [21,22]. In addition, research suggests that PsyCap reduces job burnout, depressive symptoms, occupational stress and other negative conditions. For example, PsyCap was identified as a mediator in the association between job burnout and turnover intention among Chinese nurses [23]. A previous study shows that PsyCap partially mediated the association between occupational stress and depressive symptoms for female physicians [20]. Another study found that PsyCap was a mediator in the relationship between WFC and burnout among Chinese nurses [24]. Meanwhile, another study also found that PsyCap was a moderator in the association between emotional labor, burnout, and job satisfaction [25]. Although both the relationship between PsyCap and depressive symptoms and the relationship between WFC and depressive symptoms have been investigated in previous studies [12,26], no sufficient studies demonstrated that PsyCap worked as a mediator or moderator in the association between WFC and depressive symptoms for Chinese female nurses.

Each of the four components of PsyCap has different characteristics. Self-efficacy is the ability to believe that one can take on challenge tasks and complete them; optimism is an explanatory style about positive self-attribution; hope is a positive motivational state about progressing from paths to goals at any time; and resiliency is a self-regulatory state that allows one to bounce back from difficult positions and adversity [22]. The study shows that the four components of PsyCap were negatively related with WFC [27], and another study shows that the four components of PsyCap were negatively associated with depressive symptoms [28]. In addition, a previous study found that resilience and optimism partially mediated the relationship between perceived organizational support and depressive symptoms in frontline correctional officers [28]. Meanwhile, another study shows that self-efficacy was a moderator in the relationship between WFC and job stress [29]. Thus, we hypothesize that the four components of PsyCap work as a mediator or moderator in the relationship between WFC and depressive symptoms for Chinese female nurses.

The present study has two aims. First, we will test the relationship between WFC and depressive symptoms among Chinese nurses. Second, we will examine the mediating and moderating role of PsyCap and the dimensions of PsyCap in the association between WFC and depressive symptoms.

## 2. Methods

### 2.1. Subjects and Data Collection

A cross-sectional study was carried out in Shenyang, Liaoning Province, during 2013. Based on the geographic division of Shenyang, the whole city consists of five regions (eastern, western, southern, northern, and central). Two large polyclinics (>500 beds) were randomly selected in each sampled region, with a total of 10 large polyclinics. Because male nurses only accounted for <1% of Chinese nurses [30], we just pay attention to female nurses in this study. After obtaining the informed consent to launch an investigation, a self-administered questionnaire was distributed to the nurses. The questionnaire was sent to 1100 recruited nurses and was returned by 824 of them (effective response rate: 74.9%).

The procedures followed were in accordance with the ethical standards of the Committee on Human Experimentation of China Medical University.

### 2.2. Demographic Characteristics

Three demographic characteristics obtained were age, education and marital status. Age was classified as ≤30, 31~39, and ≥40. Education was classified as professional school, junior college or college or above. Marital status was classified as single and married/cohabitation. 

### 2.3. Measurement of Depressive Symptoms

The questionnaire of the Center for Epidemiologic Studies Depression Scale (CES-D) was selected to assess the depressive symptoms of nurses [31,32]. The Chinese version of the questionnaire had good reliability and validity, and it was widely used among Chinese populations [20]. Each item included four feasible responses: (0) never; (1) sometimes; (2) frequently; and (3) always, with a total score of 60. The CES-D includes four factors: depressed affect (blue, depressed, lonely, cry, sad—items 1, 3, 6, 9, 10, 14, 17, 18); positive affect (good, hopeful, happy, enjoy—items 4, 8, 12, 16); somatic and retarded activity (bothered, appetite, effort, sleep, get going—items 2, 5, 7, 11, 13, 20); interpersonal (unfriendly, dislike—items 15 and 19). The CES-D measures symptoms during the week previous to the administration of the questionnaire. The standard CES-D Scale employs a cutoff of 16 points for depressive symptoms (CES-D ≥ 16). In this study, Cronbach’s alpha for the CES-D was 0.895.

### 2.4. Measurement of Work-Family Conflict and Family-Work Conflict

Work-family conflict was measured by two scales: the WFC scale and FWC scale [11]. The WFC scale measures the extent to which work interferes with family, while the FWC scale measures the extent to which family interferes with work. The total scale consists of 18 items, and both dimensions are measured by nine items. Each of the items is scored on a Likert scale in which 1 indicates never and 5 indicate always. We summed responses for both scales (WFC and FWC) and calculated the results to get an average score for WFC and FWC, respectively. Higher values indicate higher levels of WFC and FWC. Results of previous studies demonstrated good reliability and validity of the Chinese version of the work-family scale [33,34]. In the present study, the Cronbach’s alpha coefficients of WFC and FWC were 0.796 and 0.903.

### 2.5. Measurement of PsyCap

PsyCap was measured using the Psychological Capital Questionnaire (PCQ-24), which was developed by Luthans *et al*. [35]. The PCQ-24 consists of four dimensions: self-efficacy, hope, resilience and optimism. The total scale consists of 24 items, and each of the four dimensions is measured by six items. Each of the items is scored on a 6-point Likert scale in which 1 indicates strongly disagree and 6 indicates strongly agree. All questions ask the participants how they “feel right now.” Higher values indicate higher levels of experienced PsyCap. The Chinese version of the PCQ-24 has been used in Chinese studies, and it has demonstrated satisfactory reliability and validity [36,37]. In the present study, the Cronbach’s alpha coefficients of self-efficacy, hope, resilience, optimism and PsyCap were 0.855, 0.854, 0.802, 0.793 and 0.890, respectively, for nurses. In this study, we use two models to test the mediation of PsyCap: the first tests the four dimensions of PsyCap’s mediating role, the second tests the mediating role of the overall psychological capital instead of each construct of PsyCap. Responses for the 24 questions were averaged to get an average score as the indicator for overall PsyCap.

### 2.6. The Validities for the Scales

**Table 1:** The validities for the scales.

Model	GFI	AGFI	NFI	TLI	CFI	RMSEA
Standard	>0.9	>0.8	>0.8	>0.9	>0.9	<0.08
WFC	0.985	0.968	0.985	0.983	0.990	0.046
FWC	0.987	0.971	0.990	0.990	0.994	0.040
PsyCap	0.919	0.891	0.914	0.917	0.933	0.063
Self-efficacy	0.990	0.957	0.988	0.969	0.990	0.072
Hope	0.990	0.966	0.988	0.976	0.990	0.063
Resilience	0.996	0.986	0.994	0.993	0.997	0.033
Optimism	1.000	0.999	1.000	1.004	1.000	0.000
Depressive symptoms	0.909	0.882	0.907	0.910	0.924	0.069

### 2.7. Statistical Analysis

We used SPSS 17.0 to analyze data. Study variables need to be compared among age groups, education groups, and marital status groups by *t*-test and one-way ANOVA analyses. All statistical tests were two-sided (α = 0.05).

All the continuous variables including the predictor variables and the moderating variables were centralized to test the moderating role before regression analyses were performed. [38]. In addition, we used tolerance and variance inflation factors to check for multicollinearity. Pearson’s correlation coefficients were used to examine correlations among continuous variables. Hierarchical linear regression analyses were used to explore the moderation of PsyCap in the relationship between WFC and depressive symptoms. In step 1 of the hierarchical linear regression analyses, the control variables, age, marital status and education were used as predictors because they were assumed to be related to study variables. Two variables were set as dummy variables, due to marital status and education being categorical variables. For marital status, “single” was set as the reference group. For education, “Junior college” was set as the reference group. In step 2, WFC or FWC scales and PsyCap were added. In step 3, WFC × PsyCap or FWC × PsyCap was added. 

**Figure 1:**
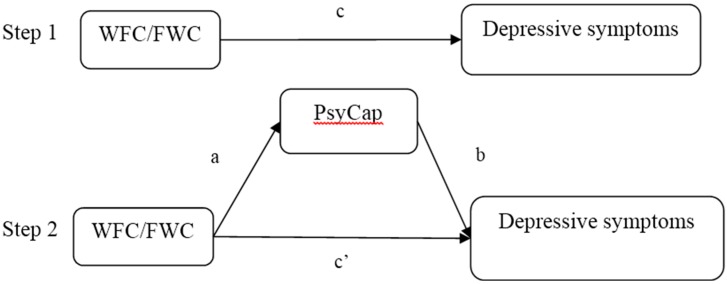
Theoretical model of the mediation of PsyCap on the association between work-family conflict and depressive symptoms. (**c**) the associations of WFC or FWC with depressive symptoms; (**a**) the relationship between work-family conflict and PsyCap; (**b**) the association of PsyCap with depressive symptoms after controlling for the independent variables; (**c’**) the relationship between work-family conflict and depressive symptoms after adding PsyCap as a mediator.

Asymptotic and resampling strategies were used to examine PsyCap as a potential mediator of the association between WFC and depressive symptoms [39]. WFC or FWC scales were modeled as independent variables, with depressive symptoms as the outcomes, PsyCap and its components as a mediator (as shown in Figure 1), and age, marital status and education as covariates. In the first step, the aim is to identify the relationship between WFC/FWC and depressive symptoms (the c path) and the second step in the analysis is to examine the mediation of PsyCap (the a × b path). If the c’ path coefficient in the second step was smaller than the c path coefficient in the first step, or was not significant, the mediation of PsyCap may exist. Five thousand bootstrap samples were use to estimate the presented study. A bias-corrected and accelerated 95% confidence interval (BCa 95% CI) was determined for each a × b product, and a BCa 95% CI excluding 0 indicated significant mediation.

## 3. Result

### 3.1. Participant Characteristics

Demographic characteristic of subjects and distributions of depressive symptoms were shown in Table 2. The mean score of young people in this study was significantly higher than that of old ones in depressive symptoms. Compared with other age groups, people less than 40 years old were more likely to have depressive symptoms. In the marital status group, there was a difference in scores for depressive symptoms. The scores of those who had married were lower than others.

**Table 2:** Demographic characteristic of subjects and the distributions of depressive symptoms.

Variable	N (%)	Depressive Symptoms
		Mean (SD)
Age		
≤30	322 (39.1%)	20.38 (11.04) **
31–39	252 (30.6%)	20.09 (10.32) **
≥40	250 (30.3%)	16.50 (9.49)
Education		
High school or under	147 (17.84%)	17.42 (9.50)
Junior college	484 (58.74%)	19.88 (11.14) *
Undergraduate or above	193 (23.42%)	18.48 (9.37)
Marital Status		
Single	218 (26.5%)	20.86 (11.34) **
Married/Cohabitation	606 (73.5%)	18.48 (10.11)

SD: standard deviations, * *p* < 0.05, ** *p* < 0.01.

### 3.2. Correlations between Study Variables

Table 3 has shown the results of the Pearson correlation. Both WFC and FWC were significantly and positively associated with depressive symptoms. However, the effects of WFC and FWC on each dimension of PsyCap were different. WFC and FWC were negatively related with hope, resilience and optimism. FWC had a negative impact on self-efficacy (Table 3).

**Table 3:** Means, standard deviations (SD) and correlations of continuous variables.

Variables	Mean	SD	1	2	3	4	5	6	7	8
1. Age	34.88	8.87								
2. Depressive symptoms	19.11	10.50	−0.163 **							
3. WFC	3.35	0.71	−0.094 **	0.249 **						
4. FWC	2.51	0.79	−0.134 **	0.437 **	0.420 **					
5. Self-efficacy	4.38	0.82	0.105 **	−0.225 **	0.077 *	−0.098 **				
6. Hope	4.14	0.87	0.071 *	−0.307 **	−0.081 *	−0.081 *	0.618 **			
7. Resilience	4.27	0.78	0.158 **	−0.324 **	−0.081 *	−0.173 **	0.531 **	0.602 **		
8. Optimism	4.28	0.90	0.151 **	−0.326 **	−0.070 *	−0.155 **	0.461 **	0.550 **	0.636 **	
9. Psychological capital	4.17	0.60	0.151 **	−0.414 **	−0.084 *	−0.209 **	0.798 **	0.856 **	0.798 **	0.714 **

* *p* < 0.05, ** *p* < 0.01.

### 3.3. PsyCap and Its Components as the Moderating Role: WFC as the Independent Variable in Regression Analysis

As shown in Table 4, after adding the control variables, WFC was positively correlated with depressive symptoms (β = 0.216, *p* < 0.01), but PsyCap was negatively correlated with depressive symptoms (β = −0.388, *p* < 0.01). The interaction of WFC and PsyCap had a significant positive association with depressive symptoms (β = 0.062, *p* < 0.05). PsyCap had a positive moderation affect in the association between WFC and depressive symptoms (β = 0.062, *p* < 0.05).

**Table 4:** WFC as the independent variable and PsyCap as the moderator in the regression analysis.

Variable	Depressive Symptoms					
	Step1 (β)	VIF	Step2 (β)	VIF	Step3 (β)	VIF
Age	−0.155 **	1.723	−0.042	1.786	−0.041	1.787
Dummy_marry1	0.004	1.675	−0.052	1.691	−0.052	1.691
Dummy_education1	−0.057	1.126	−0.060	1.132	−0.061	1.132
Dummy_education2	−0.052	1.089	−0.075 *	1.100	−0.073 *	1.101
WFC			0.216 **	1.039	0.216 **	1.039
PsyCap			−0.388 **	1.036	−0.381 **	1.049
WFC × PsyCap					0.062 *	1.015
R2	0.031		0.233		0.237	
ΔR2	0.031 **		0.202 **		0.004 *	

Notes: Dummy_marry1 means “Single” *vs.* “Married/Cohabitation”; Dummy_education1 mean “High school or under” *vs.* “Junior college”, Dummy_education2 means “Undergraduate or above” *vs.* “Junior college”. VIF means variance inflation factor. * *p* < 0.05, ** *p* < 0.01 (2-tailed).

As shown in Table 5 and Table 6, after adding the control variables, WFC was positively correlated with depressive symptoms (β = 0.263, *p* < 0.01), but self-efficacy or hope was negatively associated with depressive symptoms (β = −0.240, *p* < 0.01). The interaction of WFC and self-efficacy and the interaction of WFC and hope were significantly associated with depressive symptoms (β = 0.154, *p* < 0.05; β = 0.090, *p* < 0.05). Self-efficacy or hope had a positively moderating effect in the relationship between WFC and depressive symptoms (β = 0.154, *p* < 0.05). As shown in Table 7 and Table 8, the interaction of WFC and resilience and the interaction of WFC and optimism had no significantly relationship with depressive symptoms. Resilience and optimism did not moderate the relationship between WFC and depressive symptoms.

**Table 5:** WFC as the independent variable and self-efficacy as the moderator in the regression analysis.

Variable	Depressive Symptoms					
	Step1 (β)	VIF	Step2 (β)	VIF	Step3 (β)	VIF
Age	−0.155 **	1.723	−0.075	1.784	−0.076	1.784
Dummy_marry1	0.004	1.675	−0.043	1.696	−0.042	1.696
Dummy_education1	−0.057	1.126	−0.066	1.414	−0.069 *	1.414
Dummy_education2	−0.052	1.089	−0.073 *	1.100	−0.069 *	1.101
WFC			0.263 **	1.041	0.256 **	1.043
Self-efficacy			−0.240 **	1.037	−0.214 **	1.064
WFC × Self-efficacy					0.154 *	1.030
R2	0.031		0.143		0.166	
ΔR2	0.031**		0.112 **		0.023 **	

Notes: Dummy_marry1 means “Single” *vs.* “Married/Cohabitation”; Dummy_education1 mean “High school or under” *vs.* “Junior college”, Dummy_education2 means “Undergraduate or above” *vs.* “Junior college”. VIF means variance inflation factor. * *p* < 0.05, ** *p* < 0.01 (2-tailed).

**Table 6:** WFC as the independent variable and hope as the moderator in the regression analysis.

Variable	Depressive Symptoms					
	Step1 (β)	VIF	Step2 (β)	VIF	Step3 (β)	VIF
Age	−0.155 **	1.723	−0.090 *	1.754	−0.089	1.754
Dummy_marry1	0.004	1.675	−0.036	1.687	−0.033	1.688
Dummy_education1	−0.057	1.126	−0.056	1.132	−0.057 *	1.132
Dummy_education2	−0.052	1.089	−0.083 *	1.101	−0.082 *	1.101
WFC			0.222 **	1.039	0.227 **	1.042
Hope			−0.285 **	1.016	−0.291 **	1.019
WFC × Hope					0.090 **	1.009
R2	0.031		0.168		0.176	
ΔR2	0.031 **		0.137 **		0.008 **	

Notes: Dummy_marry1 means “Single” *vs.* “Married/Cohabitation”; Dummy_education1 mean “High school or under” *vs.* “Junior college”, Dummy_education2 means “Undergraduate or above” *vs.* “Junior college”. VIF means variance inflation factor. * *p* < 0.05, ** *p* < 0.01 (2-tailed).

**Table 7:** WFC as the independent variable and resilience as the moderator in the regression analysis.

Variable	Depressive Symptoms					
	Step1 (β)	VIF	Step2 (β)	VIF	Step3 (β)	VIF
Age	−0.155 **	1.723	−0.070	1.776	−0.071	1.777
Dummy_marry1	0.004	1.675	−0.035	1.686	−0.033	1.687
Dummy_education1	−0.057	1.126	−0.033	1.131	−0.031	1.131
Dummy_education2	−0.052	1.089	−0.065	1.102	−0.062	1.103
WFC			0.224 **	1.039	0.221 **	1.041
Resilience			−0.290 **	1.035	−0.291 **	1.035
WFC × Resilience					0.057	1.004
R2	0.031		0.169		0.172	
ΔR2	0.031 **		0.138 **		0.003	

Notes: Dummy_marry1 means “Single” *vs.* “Married/Cohabitation”; Dummy_education1 mean “High school or under” *vs.* “Junior college”, Dummy_education2 means “Undergraduate or above” *vs.* “Junior college”. VIF means variance inflation factor. * *p* < 0.05, ** *p* < 0.01 (2-tailed).

**Table 8:** WFC as the independent variable and optimism as the moderator in the regression analysis.

Variable	Depressive Symptoms					
	step1 (β)	VIF	step2 (β)	VIF	step3 (β)	VIF
Age	−0.155 **	1.723	−0.063	1.783	−0.062	1.785
Dummy_marry1	0.004	1.675	−0.043	1.691	−0.044	1.692
Dummy_education1	−0.057	1.126	−0.042	1.130	−0.043	1.130
Dummy_education2	−0.052	1.089	−0.077 *	1.100	−0.076	1.101
WFC			0.228 **	1.037	0.228 **	1.037
Optimism			−0.299 **	1.032	−0.299 **	1.032
WFC × Optimism					0.029	1.002
R2	0.031		0.175		0.175	
ΔR2	0.031 **		0.134 **		0.001	

Note: Dummy_marry1 means “Single” *vs.* “Married/Cohabitation”; Dummy_education1 mean “High school or under” *vs.* “Junior college”, Dummy_education2 means “Undergraduate or above” *vs.* “Junior college”. VIF means variance inflation factor. * *p* < 0.05, ** *p* < 0.01 (2-tailed).

### 3.4. PsyCap as the Moderating Role: FWC as the Independent Variable in Regression Analysis

As shown in Table 9, after adding the control variables, FWC was positively associated with depressive symptoms (β = 0.356, *p* < 0.01), and PsyCap was negatively associated with depressive symptoms (β = −0.333, *p* < 0.01). However, the interaction of FWC and PsyCap was not significantly correlated with depressive symptoms. PsyCap did not moderate the relationship between FWC and depressive symptoms.

**Table 9:** FWC as the independent variable and PsyCap as the moderator in the regression analysis.

Variable	Depressive Symptoms					
	Step1 (β)	VIF	Step2 (β)	VIF	Step3 (β)	VIF
Age	−0.155 **	1.723	−0.042	1.778	−0.043	1.780
Dummy_marry1	0.004	1.675	−0.028	1.686	−0.027	1.686
Dummy_education1	−0.057	1.126	−0.039	1.138	−0.038	1.140
Dummy_education2	−0.052	1.089	−0.052	1.089	−0.051	1.090
FWC			0.356 **	1.068	0.355 **	1.071
PsyCap			−0.333 **	1.072	−0.331 **	1.078
FWC×PsyCap					0.025	1.012
R2	0.031		0.308		0.308	
ΔR2	0.031 **		0.277 **		0.001	

Notes: Dummy_marry1 means “Single” *vs.* “Married/Cohabitation”; Dummy_education1 mean “High school or under” *vs.* “Junior college”, Dummy_education2 means “Undergraduate or above” *vs.* “Junior college”. VIF means variance inflation factor. * *p* < 0.05, ** *p* < 0.01 (2-tailed).

### 3.5. PsyCap as the Mediating Role: WFC as the Independent Variable in the Regression Analysis

As shown in Table 10, in step 2, WFC was positively associated with depressive symptoms (β = 0.243, *p* < 0.01). In step 3 model 1, three dimensions of PsyCap (hope, resilience, optimism) were significantly and negatively associated with depressive symptoms (β = −0.118, *p* < 0.01; β = −0.101, *p* < 0.05; β = −0.153, *p* < 0.01). In step 3 model 2, PsyCap was significantly and negatively associated with depressive symptoms (β = −0.388, *p* < 0.01). The effect of WFC on depressive symptoms in step 3 was reduced compared with that in step 2, when the dimensions of PsyCap or overall PsyCap were added (β = 0.223, *p* < 0.01; β = 0.216, *p* < 0.01). Thus, the results suggest that hope, resilience, and optimism may have a partially mediating effect in the relationship between WFC and depressive symptoms when they are added. Overall PsyCap may have a partially mediating effect in the relationship between WFC and depressive symptoms as well.

**Table 10:** Hierarchical linear regression analysis results of WFC.

Variable	Depressive Symptoms							
	Step1 (β)	VIF	Step2 (β)	VIF	Step3 (β)			
					model1	VIF	model2	VIF
Age	−0.155 **	1.723	−0.124 **	1.740	−0.052	1.807	−0.042	1.786
Dummy_marry1	0.004	1.675	−0.015	1.682	−0.049	1.699	−0.052	1.691
Dummy_education1	−0.057	1.126	−0.042	1.130	−0.049	1.152	−0.060	1.132
Dummy_education2	−0.052	1.089	−0.077 *	1.100	−0.074 *	1.106	−0.075 *	1.100
WFC			0.243 **	1.034	0.223 **	1.073	0.216 **	1.039
Self-efficacy					−0.041	1.832		
Hope					−0.118 **	2.080		
Resilience					−0.101 *	2.103		
Optimism					−0.153 **	1.840		
Psychological capital							−0.388 **	1.036
R2	0.031		0.088		0.203		0.233	
ΔR2	0.031 **		0.057 **		0.115 **		0.145 **	

Notes: Dummy_marry1 means “Single” *vs.* “Married/Cohabitation”; Dummy_education1 mean “High school or under” *vs.* “Junior college”, Dummy_education2 means “Undergraduate or above” *vs.* “Junior college”. VIF means variance inflation factor. * *p* < 0.05, ** *p* < 0.01.

As shown in Table 11, the association between WFC and depressive symptoms (the c path) was examined. WFC had a positive correlation with depressive symptoms. The mediation of PsyCap on the association between WFC and depressive symptoms was then estimated.

**Table 11:** WFC as the independent variable and PsyCap as the mediator in the regression analysis.

	Mediators	c	a	b	c’	a × b (BCa 95% CI)
*N* = 824	PsyCap	0.243 **	−0.070 *	−0.388 **	0.216 **	0.027 (−0.002, 0.059)

Notes: c: correlation of WFC with depressive symptoms; a: correlation of WFC with PsyCap; b: correlation of PsyCap with depressive symptoms after controlling for the covariate; c’: the association between WFC and depressive symptoms after adding PsyCap as a mediator.; a × b: the product of a and b; BCa 95% CI: the bias-corrected and accelerated 95% confidence interval age, education and marital status; * *p*<0.05, ** *p*<0.01 (2-tailed).

For nurses, WFC was negatively correlated with PsyCap (the a path). PsyCap has a significant and negative association with depressive symptoms after controlling for WFC (the b path), but a BCa 95% CI including 0 indicates that PsyCap did not play a significant mediation role in this association. Thus, PsyCap did not mediate the association between WFC and depressive symptoms.

### 3.6. PsyCap and Its Components as the Mediating Role: FWC as the Dependent Variable in the Regression Analysis

As shown in Table 12, in step 2, FWC was positively associated with depressive symptoms (β = 0.422, *p* < 0.01). In step 3 model 1, two dimensions of PsyCap (hope, optimism) were significantly and negatively associated with depressive symptoms (β = −0.175, *p* < 0.01; β = −0.126, *p* < 0.01). In step 3 model 2, PsyCap was significantly and negatively associated with depressive symptoms (β = −0.333, *p* < 0.01). The effect of FWC on depressive symptoms in step 3 was reduced when compared with that of step 2, when the dimensions of PsyCap or overall PsyCap were added (β = 0.382, *p* < 0.01; β = 0.356, *p* < 0.01). Thus, the results suggest that hope and optimism may have a partially mediating effect in the relationship between FWC and depressive symptoms when they are added. Overall PsyCap may have a partially mediating effect in the relationship between FWC and depressive symptoms.

**Table 12:** Hierarchical linear regression analysis results of FWC.

Variable	Depressive Symptoms							
	Step1 (β*)*	VIF	Step2(β)	VIF	Step3 (β)			
					model1	VIF	model2	VIF
Age	−0.155 **	1.723	−0.108 **	1.736	−0.054	1.794	−0.042	1.778
Dummy_marry1	0.004	1.675	0.006	1.675	−0.023	1.691	−0.028	1.686
Dummy_education1	−0.057	1.126	−0.018	1.134	−0.025	1.159	−0.039	1.138
Dummy_education2	−0.052	1.089	−0.051	1.089	−0.054	1.096	−0.052	1.089
FWC			0.422 **	1.026	0.382 **	1.056	0.356 **	1.068
Self-efficacy					0.023	1.778		
Hope					−0.175 **	2.060		
Resilience					−0.072	2.112		
Optimism					−0.126 **	1.847		
Psychological capital							−0.333 **	1.072
R^2^	0.031		0.204		0.295		0.308	
ΔR^2^	0.031 **		0.173 **		0.090 **		0.103 **	

Notes: Dummy_marry1 means “Single” *vs.* “Married/Cohabitation”; Dummy_education1 mean “High school or under” *vs.* “Junior college”, Dummy_education2 means “Undergraduate or above” *vs.* “Junior college”. VIF means variance inflation factor. * *p* < 0.05, ** *p* < 0.01.

As shown in Table 13, the association between FWC and depressive symptoms (the c path) was examined. FWC had a positively relation with depressive symptoms. The mediation of PsyCap and its components on the association between FWC and depressive symptoms was then estimated.

For nurses, FWC has a negative association with PsyCap, self-efficacy, hope and optimism (the a path). PsyCap, hope and optimism have significant and negative correlations with depressive symptoms after controlling for FWC (the b path), but self-efficacy and resilience were not significantly correlated with depressive symptoms after controlling for FWC (the b path). Thus, PsyCap, hope and optimism significantly mediated the association between FWC and depressive symptoms. The direct pathways between FWC and depressive symptoms (the c’ path) remained significant when PsyCap, hope or optimism was added in the model as a mediator.

We calculated the proportion of the total effect of the independent variable on the outcome variable (c) that was mediated by PsyCap, hope and optimism using the formula (a × b)/c, and the reason is to determine the effect size of the mediating pathway. The proportion of PsyCap mediation was 18.25% for FWC, the proportion of hope mediation was 3.08% for FWC, and the proportion of optimism mediation was 4.03% for FWC.

**Table 13:** FWC as the independent variable and PsyCap and its components as mediators in the regression analysis.

	Mediators	c	a	b	c’	a × b (BCa 95% CI)
*N* = 824	PsyCap	0.356 **	−0.197 **	−0.333 **	0.422 **	0.065 (0.043, 0.094)
	Self-efficacy	0.422 **	−0.096 **	0.023	0.382 **	−0.002 (−0.013, 0.005)
	Hope	0.422 **	−0.076 *	−0.175 **	0.382 **	0.013 (0.002, 0.028)
	Resilience	0.422 **	−0.153 **	−0.072	0.382 **	0.011 (−0.001, 0.030)
	Optimism	0.422 **	−0.138 **	−0.127 **	0.382 **	0.017 (0.006, 0.038)

Notes: c: correlation of FWC with depressive symptoms; a: correlation of FWC with PsyCap; b: correlation of PsyCap, self-efficacy, hope, resilience and optimism with depressive symptoms after controlling for the covariate; c’: correlation of FWC with depressive symptoms after adding PsyCap, self-efficacy, hope, resilience and optimism as a mediator.; a × b: the product of a and b; BCa 95% CI: the bias-corrected and accelerated 95% confidence interval age, education and marital status; * *p* < 0.05, ** *p* < 0.01 (2-tailed).

## 4. Discussion

In this study, subjects were selected from hospitals in Shenyang, China. A large number of samples and a high effective response rate show a good representation of our study population, and they could enhance the universalization of the conclusion in our study. Regarding the association between work-family conflict and depressive symptoms, in the present study, WFC and FWC were found to be positively related to depressive symptoms. Similar results can be found in previous studies [10,11,12,40]. One of the explanations is that work-related factors, poor relationships in the work environment, and work insecurity had a significant relationship with symptoms of depression [41]. Another explanation is that high levels of FWC destroy one’s ability to perform effectively at work and one’s work-related self-image may be undermined, which in turn may cause psychological distress [42]. Hospital administrators should be aware of the risk of work-family conflict demonstrated by these findings. It can be concluded that WFC has the potential to affect the quality of work. Some interventions should be made to decrease nurses’ WFC and FWC and to reduce nurses’ depressive symptoms. As an example, a better working environment and teamwork should be encouraged by measures of organizational development in hospitals [11,12,24]. In addition, nurses’ working hours are long and provide little flexibility, thus, hospital administrators could adjust nurses’ working hours to reduce the level of work-family conflict. Meanwhile, hospital administrators should increase the number of staff and give nurses more chances to improve their technical skills to better accommodate a high-tech environment and to decrease the level of work-family conflict.

As an identified positive resource, PsyCap can decrease job stress and burnout in the workplace [43,44] and help counteract depressive symptoms in employees [45]. In this study, PsyCap was found to be negatively related to depressive symptoms among Chinese female nurses. This finding shows that PsyCap had an effect on depressive symptoms and was a positive resource for fighting against depressive symptoms among this demographic. To our knowledge, PsyCap was identified as a mediator or moderator in some relationships [20,23,24]. This study is the first one to explore the mediation and moderation of PsyCap and its dimensions in the association between work family conflict and depressive symptoms in China.

For female nurses, our study demonstrated that PsyCap partially mediated in the association between FWC and depressive symptoms. This suggests that FWC can be harmful to PsyCap, and reduce the level of nurses’ PsyCap, so as to lead to nurses’ depressive symptoms. Regarding the PsyCap components, hope and optimism partially mediated the effects of FWC on depressive symptoms. This result suggests that FWC can be harmful to hope and optimism, and may also lead to nurses’ depressive symptoms. Self-efficacy doesn’t mediate the effects of FWC on depressive symptoms. Nurses may work rigorous and taxing schedules and they may still not satisfy work demands and expectations. Therefore, the effect of self-efficacy does not work in mediating these relationships. On the other hand, it could be that low levels of FWC arise from daily events that may be more easily controlled by highly efficacious individuals. However, high levels of FWC may arise from more serious events that are less controllable or are perceived as less controllable [46,47]. Lastly, people who are high in self-efficacy may become disappointed by trying to control things that simply cannot be controlled. 

As a product of psychological capacities, PsyCap has an impact on depressive symptoms and its mediation on the association between FWC and depressive symptoms was greater than the effects of PsyCap components among nurses. This result is in accordance with a previous study that indicated the synergistic role of PsyCap components [45]. Thus, it is necessary to focus and invest more resources in significant components than non-significant components, and achieve maximum positive outcomes.

In addition, in this study, PsyCap had a positive moderation in the association between WFC and depressive symptoms. For the nurses with a high level of PsyCap, depressive symptoms will be increased with the increase of WFC. However, for the nurses with the low level of PsyCap, with the increase of WFC, depressive symptoms will not be obviously increased. This suggests that hospital managers should improve nurses’ levels of PsyCap, which plays a positive role, while some measures should be taken to reduce the level of WFC or the positive effects of PsyCap will be subject to a certain degree of damage. Among the PsyCap components, self-efficacy and hope had positively moderating effects in the association between WFC and depressive symptoms. The results show that the self-efficacy and hope of PsyCap play a major role in this relationship. This suggests that hospital managers should control the investment in self-efficacy and hope of PsyCap while reducing the level of WFC.

Compared to reducing nurses’ work-family conflict, it is a more effective and feasible strategy for general hospitals to develop resources increasing the PsyCap of nurses. Some interventions should be taken to develop the PsyCap in China. People should specifically pay more attention to improving hope and optimism in the PsyCap investment. For hope improvement, some appropriate and challenging job goals should be set for nurses to help them strengthen their willpower. Meanwhile, managers should help nurses create multiple pathways to reach their goals. For improving optimism, managers should encourage nurses to treat past failures and setbacks as valuable experiences, build a positive attribution style, and improve their capacity to discover and pursue different opportunities [48,49,50].

## 5. Conclusions

To summarize, our findings revealed that work-family conflict is a risk factor that can increase nurses’ depressive symptoms, and PsyCap is a positive resource to combat nurses’ depressive symptoms. PsyCap as a moderator can aggravate the effects of WFC on depressive symptoms. Meanwhile, when PsyCap is a mediator, FWC impacts PsyCap to increase nurses’ depressive symptoms.

Several limitations of this study must be considered. First, this study is not a longitudinal study. Work-family conflict, PsyCap and depressive symptoms were measured simultaneously, so we cannot arrive at causal conclusions. Secondly, in this study, the target population we set is large hospital nurses, and this does not cover all groups of nurses, like nurses who work at small hospitals or community service centers. Next, we will study the nurses of these institutions. Third, this study was only concerned with the association between work-family conflict and depressive symptoms and controlled only for some basic social demographics. More risk factors and possible confounders should be investigated in further studies.
